# Editorial: Disclosure in sexual and reproductive health

**DOI:** 10.3389/fgwh.2025.1681646

**Published:** 2025-08-20

**Authors:** Pooja Chitneni, Shan Qiao, Simon M. Manga, Janet M. Turan

**Affiliations:** ^1^ Divison of Global Health Equity, Brigham and Women’s Hospital, Boston, MA, United States; ^2^ Harvard Medical School, Boston, MA, United States; ^3^ Department of Health Promotion, Education and Behavior, Arnold School of Public Health, University of South Carolina, Columbia, SC, United States; ^4^ SC SmartState Center for Healthcare Quality (CHQ), University of South Carolina, Columbia, SC, United States; ^5^ Women’s Health Program, Cameroon Baptist Convention Health Services, Bamenda, Northwest Region, Cameroon; ^6^ Division of Global and Rural Health, Department of Obstetrics & Gynecology, University of Alabama at Birmingham, Birmingham, AL, United States; ^7^ Department of Health Policy and Organization, School of Public Health, University of Alabama at Birmingham, Birmingham, AL, United States; ^8^ Department of Public Health, School of Medicine, Koc University, Istanbul, Türkiye

**Keywords:** disclosure, sexual and reproductive health, editorial, gender, power

**Editorial on the Research Topic**
Disclosure in sexual and reproductive health

Disclosure of a stigmatized identity or health condition can be difficult. Examples of identities or conditions people might not wish to disclose include one's HIV status, HIV pre-exposure prophylaxis (PrEP) use, non-HIV sexually transmitted infections (STIs), mental illness, LGBTQ + identities, and many more. People may need or want to disclose for various reasons, ranging from wanting to prevent disease transmission to obtaining social support for navigating a stigmatized condition (1). Disclosure is further complicated when related to sexual and reproductive health, which often layers in unequal gender power dynamics and additional forms of stigma. For example, in heterosexual relationships, women often have less relationship power compared to men, which may complicate disclosure (2). While some models and tools exist to support the disclosure of a stigmatized identity (3–7), in general, little is known about how best to support people in this endeavor. In this Frontiers Research Collection, we collated research related to the disclosure of a stigmatized identity or condition related to sexual and reproductive health. Our goal was to gather seemingly disparate disclosure topics, that in reality share core principles, to allow researchers to build upon each other's work. The seven articles in this collection span a wide array of topics, settings, and methods that cumulatively enhance our understanding of disclosure related to sexual and reproductive health.

Two articles assessed the disclosure of a stigmatized health condition and examined correlations with disclosure recipient characteristics and reactions. Halim et al. conducted a cohort study following pregnant women in Maharashtra, India, to examine the effect of sickle cell disease (SCD) screening on intimate partner violence (IPV). In India and similar settings, positive SCD screening requires partner disclosure to facilitate paternal SCD screening to determine the likelihood of the child inheriting sickle cell trait compared to sickle cell anemia. Of the 182 participants, 91 had positive sickle cell disease screening results and 91 had negative sickle cell disease screening results. Participants with positive SCD screening results were ≥2 times as likely to experience IPV compared to participants with negative SCD screening results. Mulawa et al. assessed HIV disclosure among adolescents with perinatally-acquired HIV (APHIV) using egocentric social network analysis. The team analyzed social network data from 58 APHIV (mean age 14 years) with 349 relationships. They found that participants disclosed their HIV status to only one-third of their relationships. Using multivariable regression, they found significant increases in HIV disclosure odds when participants presumed the potential disclosure recipient to be older than the participant, also living with HIV, and trustworthy.

Articles in this collection also used qualitative methods to explore questions around disclosure related to PrEP and STIs. Beesham et al. conducted in-depth, qualitative interviews with 13 women aged 18–25 years who had participated in the Evidence for Contraceptive Options and HIV Outcomes (ECHO) Trial in Durban, South Africa, and who accessed and continued PrEP through study exit, to explore aspects of oral PrEP disclosure. They found that all participants disclosed PrEP use to at least one disclosure recipient who was usually a family member and/or sexual partner. PrEP disclosure led to increased PrEP adherence support from disclosure recipients. However, some women reported barriers to disclosure related to PrEP stigma and misconceptions. Mwima et al. similarly explored PrEP disclosure barriers through in-depth, qualitative interviews with 14 women aged 18–24 years on PrEP for ≥6 months who had recently engaged in transactional sex in Kampala, Uganda. They found that PrEP disclosure barriers included PrEP stigma and misconceptions, fear of judgment and violence from disclosure recipients, and a lack of disclosure guidance from healthcare institutions. Strategies to overcome these barriers included utilizing psychosocial coping skills, disclosing to peers for social support, and connecting with healthcare workers for PrEP information and disclosure support. Finally, Chitneni et al. explored STI partner notification (PN) through 32 in-depth, qualitative interviews with STI stakeholders including patients with prior STI, healthcare workers, pharmacists, and healthcare administrators in rural, southwestern Uganda. Three candidate STI PN models to guide the interviews. Overall, participants found the nursing model favorable as nurses are generally trained to support STI care. The pharmacy model had potential as patients often seek pharmacy care early and as monetary incentives could align with STI PN. However, pharmacists would need STI PN training, and pharmacies would need restructuring to allow privacy. The community health worker model was deemed appropriate for education and advocacy and less so for STI treatment.

The final two manuscripts addressed interventions to reduce stigma and support HIV disclosure. Chenneville et al. described a stigma-reduction intervention (Healthy, Open, Proud) originally created for mental health and outlined the steps needed to adapt this to HIV with a specific focus on women and disclosure support. Li et al. conducted a cluster randomized controlled trial evaluating an intervention to assist adults with HIV to disclose their HIV status to household children in Guangxi, China. The team used a first-order Markov Chain Model to evaluate participants' transition through the Health Action Process Approach framework stages from pre-intention to action over 12 months. Among the 791 participants, the intervention significantly facilitated participant transition from no intention to disclose pre-intervention to actual disclosure post-intervention.

The manuscripts in this collection span the spectrum from identifying disclosure correlates to qualitatively exploring disclosure questions to developing and testing interventions. While several manuscripts focused on topics typically associated with disclosure, such as HIV, Halim et al.'s work examining associations of sickle cell disease disclosure with IPV reminds us that stigma can be associated with numerous sexual and reproductive health conditions, especially in the context of unequal gender power dynamics. Through this Collection, we found consistent themes across disclosure topics. Barriers to disclosure were often related to perceived stigma and fears of enacted stigma, such as IPV. Participants who did disclose their condition often limited their disclosure to trusted recipients who could provide social support. Additionally, Chenneville et al. outlined methods for adapting an evidence-based disclosure intervention from mental health to HIV. Our goal in this Collection is to further inspire researchers to connect and build upon similar underlying frameworks and methods to further progress the field of disclosure overall.
